# Neurocognitive predictors of treatment completion and daytime activities at follow-up in multiproblem young adults

**DOI:** 10.3758/s13415-020-00822-4

**Published:** 2020-08-20

**Authors:** M. E. Van der Sluys, J. Zijlmans, A. Popma, P. H. Van der Laan, E. J. A. Scherder, R. Marhe

**Affiliations:** 1 Department of Clinical, Neuro and Developmental Psychology, Vrije Universiteit Amsterdam, Van der Boechorstraat 7, 1081 BT Amsterdam, The Netherlands; 2 VU University Medical Center Department of Child and Adolescent Psychiatry, Meibergdreef 5, 1105 AZ Amsterdam, The Netherlands; 3 Department of Criminal Law and Criminology, Leiden University, Steenschuur 25, 2311 ES Leiden, The Netherlands; 4 Department of Criminal Law and Criminology, Vrije Universiteit Amsterdam, De Boelelaan 1105, 1081 HV Amsterdam, The Netherlands; 5 Netherlands Institute for the Study of Crime and Law Enforcement, De Boelelaan 1077, 1081 HV Amsterdam, The Netherlands; 6 Department of Psychology, Education and Child Studies, Erasmus University Rotterdam, Burgemeester Oudlaan 50, 3062 PA Rotterdam, The Netherlands

**Keywords:** Treatment outcome, Young adulthood, fMRI, EEG, Cognitive control, Antisocial

## Abstract

Previous research has shown an association between cognitive control deficits and problematic behavior such as antisocial behavior and substance use, but little is known about the predictive value of cognitive control for treatment outcome. The current study tests whether selected markers of baseline cognitive control predict (1) treatment completion of a day treatment program involving a combination of approaches for multiproblem young adults and (2) daytime activities a year after the start of treatment, over and above psychological, social, and criminal characteristics. We assessed individual, neurobiological, and neurobehavioral measures, including functional brain activity during an inhibition task and two electroencephalographic measures of error processing in 127 male multiproblem young adults (age 18–27 years). We performed two hierarchical regression models to test the predictive power of cognitive control for treatment completion and daytime activities at follow-up. The overall models did not significantly predict treatment completion or daytime activities at follow-up. However, activity in the anterior cingulate cortex (ACC) during response inhibition, years of regular alcohol use, internalizing problems, and ethnicity were all significant individual predictors of daytime activity at follow-up. In conclusion, cognitive control could not predict treatment completion or daytime activities a year after the start of treatment over and above individual characteristics. However, results indicate a direct association between brain activity during response inhibition and participation in daytime activities, such as work or school, after treatment. As adequate baseline inhibitory control is associated with a positive outcome at follow-up, this suggests interventions targeting cognitive control might result in better outcomes at follow-up.

Problematic behavior such as antisocial behavior and substance use is a major cause of public concern, with implications for victims, perpetrators, and society (Moffitt, 2017). There is a growing body of literature on the efficacy of interventions aimed at reducing antisocial behavior (Bennett & Gibbons, 2000; Dodge & McCourt, 2010; Frick, 2016; Lipsey & Cullen, 2007; Reid & Gacono, 2000). However, the effectiveness of these interventions varies, as overall reoffending rates among juveniles and adults remain high even after treatment (Fazel & Wolf, 2015; van der Put, Asscher, & Stams, 2016), and treatment noncompletion rates range from 20% to 40% (Rubin, Rabinovich, Hallsworth, & Nason, 2006). Treatment programs tend to be more effective if they are individually tailored (Frick, 2016; Rubin et al., 2006), such as by detecting and treating early individual markers of treatment outcome. This approach is in line with the risk–need–responsivity (RNR) model, which stresses the need for individually tailored interventions, and the specific treatment of factors known to be associated with successful reintegration of antisocial individuals (Andrews & Bonta, 2007; Andrews & Dowden, 2008; McRae, 2013; Polaschek, 2012). Therefore, more research on predictors of treatment outcome in antisocial populations is a promising approach to increasing treatment effectiveness and improving outcome results.

The biopsychosocial model offers a framework in the search for these predictors (Popma & Raine, 2006). This transactional model of antisocial behavior suggests that the interaction and joint contribution of biological, psychological, and social factors should be taken into account in the understanding and treatment of such behavior. During the past decades, research has shown that several individual characteristics are known to influence and predict treatment outcome, such as age, ethnicity, and intelligence (Andrews & Dowden, 2006); psychopathy (Sewall & Olver, 2019); psychopathology (McCarter et al., 2016); differentiating between internalizing and externalizing psychopathology (Winters, Stinchfield, Latimer, & Stone, 2008); aggression (Blader et al., 2013); impulsivity (Fishbein et al., 2009; Fornells, Capdevila, & Andres-Pueyo, 2002); history of delinquency (Cottle, Lee, & Heilbrun, 2001; Lipsey & Cullen, 2007); and drug abuse (Lipsey & Cullen, 2007). However, many of the current prediction models have not been approached from a biopsychosocial perspective, and thus only few studies included both individual characteristics and neurobiological measures such as functional brain activity or electrophysiological measurements (Cornet, de Kogel, Nijman, Raine, & van Der Laan, 2014; van Der Gronde, Kempes, van El, Rinne, & Pieters, 2014). Previous studies on neurobiological predictors of treatment outcomes in antisocial populations mainly focused on alterations in the autonomic nervous system (Alink et al., 2008; Beauchaine, Gartner, & Hagen, 2000; Schechter, Brennan, Cunningham, Foster, & Whitmore, 2012; van der Gronde et al., 2014). Predictors of interest have included heart rate variability (Beauchaine et al., 2000), baseline heart rate (Ortiz & Raine, 2004), skin conductance level (van der Gronde et al., 2014), cortisol levels (Alink et al., 2008; Schechter et al., 2012), and testosterone levels (Schechter et al., 2012). Increased knowledge regarding neurobiological markers of treatment outcomes in antisocial behavior could aid in the tailoring of treatment and improve rates of treatment completion (Bootsman, 2018).

A vast body of literature provides evidence for an association between antisocial behavior and cognitive control. Cognitive control (also called executive control, or executive functioning) is a multifaceted neuropsychological construct consisting of various top-down processes that are critical for goal-oriented and future-oriented behavior (Diamond, 2012; Gazzaley & D’Esposito, 2007; Sira & Mateer, 2014). It is argued that disruptions of cognitive control can lead to problematic behavior. Deficits in this higher order process could limit the possibility to adequately learn and adapt behavior in real-world situations. Various deficits in cognitive control have been associated with antisocial behavior such as impaired performance on neuropsychological measures of executive functioning (Chamberlain, Derbyshire, Leppink, & Grant, 2016; Ogilvie, Stewart, Chan, & Shum, 2011), an inability to restrain impulsive or inappropriate responses (Swann, Lijffijt, Lane, Steinberg, & Moeller, 2009; Turner et al., 2018; Weidacker, Snowden, Boy, & Johnston, 2017), an inability to detect and react to errors (Zeier, Baskin-Sommers, Hiatt Racer, & Newman, 2012), and abnormalities in neural regions associated with inhibitory control and the ability to flexibly adjust behavior (Aharoni et al., 2013; Guan et al., 2015; Sterzer, 2009; Yang & Raine, 2009; Zijlmans et al., 2019). However, little is known about the role of cognitive control as a predictor of treatment outcomes in antisocial or problematic populations. Previous research focused on the assessment of various components of executive functioning with neuropsychological tests (MicroCog: Aharonovich, Nunes, & Hasin, 2003; Stop-Change Task and Cambridge decision task: Fishbein et al., 2009; D-KEFS: Mullin & Simpson, 2007)*.* Results from these studies indicate that cognitive control can predict treatment outcome, but they did not include neurobiological measures such as functional brain activity or event-related potentials (ERPs) during cognitive-control tasks. Using a biopsychosocial approach, behavioral measures (such as reaction time and accuracy) as well as neurobiological measures should be taken into account in the prediction of antisocial behavior, in addition to individual characteristics such as age, aggression, and drug (ab)use. Previous studies in clinical populations indicate that the addition of neurobiological measures of cognitive control—specifically, two important indices: response inhibition and error processing—can aid in the prediction of substance-abuse treatment outcome or recidivism (Aharoni et al., 2014; Aharoni et al., 2013; Brewer, Worhunsky, Carroll, Rounsaville, & Potenza, 2008; Luo et al., 2013; Marhe, van de Wetering, & Franken, 2013; Paulus, Tapert, & Schuckit, 2005; Steele et al., 2014). These studies suggest that associated neural correlates such as anterior cingulate cortex (ACC) activity and error-related brain potentials might function as biomarkers for those who are vulnerable to relapse or recidivism.

Response inhibition refers to the ability to suppress inappropriate behavior (Chambers, Garavan, & Bellgrove, 2009), whereas error processing refers to the ability to detect and react to errors (Overbye et al., 2019). Both processes are critical for goal-oriented behavior in everyday life (Chambers et al., 2009; Overbye et al., 2019) and the discontinuation of maladaptive or impulsive behaviors such as substance abuse (Ivanov, Schulz, London, & Newcorn, 2008), pathological gambling (van Holst, van Holstein, van den Brink, Veltman, & Goudriaan, 2012), and aggression (Sterzer, 2009). Aberrant response inhibition manifested in behavior (i.e., shorter reaction times and more errors) has been found in antisocial populations such as child sexual offenders (Turner et al., 2018) and psychopathic offenders (Weidacker et al., 2017). Furthermore, poor error processing at the behavioral level, as measured with poor accuracy, has been related to incarcerated offenders with antisocial personality disorder (Zeier et al., 2012). In addition to behavioral measures of cognitive control, such as accuracy, reaction time, and errors, there are also neurobiological markers of cognitive control. Two neurobiological markers are event-related potentials (ERPs) and brain activity in specific regions of the (pre)frontal cortex during task performance. Converging evidence suggests activity in the ACC as a neural correlate of cognitive control (Nieuwenhuis, Yeung, van den Wildenberg, & Ridderinkhof, 2003) and specifically as a neural correlate of response inhibition (Aharoni et al., 2013; Braver, Barch, Gray, Molfese, & Snyder, 2001). Two electrophysiological indices of error processing are closely related: the error-related negativity (ERN) and error-related positivity (Pe) (Nieuwenhuis, Ridderinkhof, Blom, Band, & Kok, 2001). The ERN is a negative potential that arises approximately 25–100 ms after an incorrect response (Bernstein, Scheffers, & Coles, 1995) and is thought to reflect early, automatic error processing (Bernstein et al., 1995). In contrast, the Pe is a positive potential that follows the ERN and is thought to reflect the late, more conscious processing of an error (Luijten, van Meel, & Franken, 2011). Source localization studies have indicated that the ERN is most likely generated in the ACC and Pe amplitude has been correlated both negatively as well as positively to ACC activity (Edwards, Calhoun, & Kiehl, 2012; Orr & Hester, 2012), suggesting an important role for the ACC, ERN and Pe in cognitive control. Similarly, individuals with behavioral problems show altered activity of these specific correlates (Brazil et al., 2009; Carroll, Sutherland, Salmeron, Ross, & Stein, 2015; Rüsch et al., 2010; Steele, Maurer, Bernat, Calhoun, & Kiehl, 2016).

Regarding associations with populations demonstrating antisocial or impulsive behaviors, neuroimaging studies have shown reduced ACC activity during response inhibition and error processing in drug abusers (Hester, Nestor, & Garavan, 2009), individuals with borderline personality disorder (Rüsch et al., 2010), and smokers with greater externalizing personality traits (Carroll et al., 2015). Additionally, two previous studies showed no difference on ERN, but reduced Pe amplitude in offenders compared with healthy controls (Brazil et al., 2009; Steele et al., 2016), implying that this population has intact early (automatic) error processing but aberrant late (more conscious) processing of an error. In contrast, a recent study found no difference in Pe amplitude, but reduced ERN in multiproblem young adults compared with healthy controls, implying aberrant early error processing (Zijlmans et al., 2019; same sample as current study). Although research on response inhibition and error processing has been rapidly growing, research on their prospective association with treatment outcomes in antisocial populations is scarce and results are mixed. Some literature examines neurocognitive predictors of treatment outcomes using electrophysiological indices of error processing in substance abusers (Marhe et al., 2013; Steele et al., 2014) and offenders (Steele et al., 2017). These studies showed that in adult substance abusers, there is an association between variation in ERN and Pe amplitude and treatment outcome (Marhe et al., 2013; Steele et al., 2014). More specifically, both improved error processing (larger Pe; Steele et al., 2014) and diminished error processing (reduced ERN; Marhe et al., 2013; Steele et al., 2014) have been predictive of treatment outcomes. Additionally, real-world functioning outside of the treatment facility has also been studied. In the previously mentioned study on offenders, both reduced ACC activity and larger Pe amplitude were predictive of rearrests (Steele et al., 2017). Furthermore, prior studies have used blood-oxygen-level-dependent (BOLD) activation in the ACC to predict reoffense (Aharoni et al., 2014; Aharoni et al., 2013) where changes in the brain hemodynamic response during a response inhibition task were predictive of rearrests following prison release. More specifically, the odds of rearrest were approximately double for offenders with relatively low ACC activity compared with offenders with relatively high ACC activity. These results suggest that neurobiological measures of cognitive control are plausible predictors of treatment outcome and real-world functioning (or real-world dysfunction, such as reoffense). Despite these recent advances, the contribution of studies exploring the predictive value of neurobiological measures of cognitive control for treatment outcomes in populations displaying problematic behavior is limited, and most studies fail to apply a biopsychosocial approach.

To our best knowledge, the predictive power of neurobiological and neurobehavioral indices of cognitive control for treatment outcomes, over and above psychological, social, and criminal predictors, has not yet been studied in young adults facing multiple problems such as drug use and antisocial behavior. Furthermore, the current study is the first to include both functional magnetic resonance imaging (fMRI) activity of the ACC during response inhibition and ERP components during error processing to predict treatment outcomes. A sample of male multiproblem young adults is included at the start of multimodal (that is, involving a combination of approaches) day treatment program De Nieuwe Kans (DNK; translated as “New Opportunities”). DNK provides a multimodal day treatment program for young adults facing a range of problems—for example, a history of delinquency, behavioral and psychological problems, no daytime activities (e.g., no work, education, other full-time activities), frequent substance use, and no or low income (Luijks et al., 2017; van Duin et al., 2017; van Duin et al., 2018; Zijlmans et al., 2019). The main goal of DNK is to reintegrate participants into society by facilitating and retaining successful integration into education or employment and to increase self-sufficiency, and to subsequently reduce delinquency. The study has two aims: to test whether the selected markers of cognitive control, as measured at baseline, predict (1) treatment completion versus treatment dropout and (2) daytime activities a year after the start of treatment, over and above psychological, social, and criminal characteristics of a multimodal day treatment program in multiproblem young adults. Examples of daytime activities a year after the start of treatment are successful participation in education, work, or other full-time daytime activities such as voluntary work.

## Methods and materials

### Participants

Participants were 127 male multiproblem young adults, ranging in age from 18 to 27 years (mean age = 21.92 years, *SD* = 2.40). They were recruited at the start of the day treatment program DNK (“New Opportunities”). Eight participants were excluded because of failure to complete the tasks, six participants were excluded because fewer than six error trials were usable for analysis (Olvet & Hajcak, 2009), and eight participants did not start treatment after intake and were thus not enrolled in the multimodal day treatment program at DNK. These participants were also excluded from analysis. The final sample included 105 multiproblem young adults.

As the current study relies on previously collected data, no a priori sample size calculation could be performed for the current analysis. For the previously collected data, an a priori sample size calculation was performed that was based on a linear regression model (Zijlmans et al., 2019; Zijlmans et al., 2018), whereas in the current study, a logistic regression model is performed. With the expectation of a medium effect size, a power of 0.80, and an alpha of .05, we required a sample size of *N* = 103 for the EEG measurements (de Wied et al., 2012). Previous studies indicate a large effect size for similar fMRI-ACC measures (Fu et al., 2008; Rubia et al., 2001; van Holst et al., 2012). Thus, for the fMRI design, we performed a conservative power analysis with a medium effect size, a power of 0.80, and an alpha of .05. This resulted in a required sample size of 34 participants for the region of interest (ROI) analysis.

All procedures in the present study were in accordance with the ethical standards of the institutional and national research committee and with the 1964 Helsinki declaration and its later amendments, or comparable ethical standards. The study has been approved by the Medical Ethical Committee of the VU University Medical Center (Registration Number 2013.422–NL46906.029.13), and all participants provided written informed consent. Participants received a reimbursement of 30 euros for their participation in the fMRI and EEG protocols.

### Treatment setting

The multimodal day treatment program at DNK was specifically designed to treat young male adults (18–27 years) with severe, multiple problems (e.g., drug use, psychological problems, antisocial behavior, financial problems; van Duin et al., 2017). By applying cognitive behavioral techniques, practical support, as well as education including sports, DNK aims to improve various facets of the participants life. The main goal of DNK is the guided reintegration into society, through continued participation in education or employment or other daytime activities after treatment.

### Intervention

Participants at DNK receive group-oriented as well as individual-oriented treatment. The basis of this treatment is a multidimensional approach, in which all aspects of the participant’s life are reviewed and included in the intervention to guide them through young adulthood. The intervention focuses on treating behavioral problems—in particular, antisocial behavior, and cognitive distortions, such as self-serving (antisocial) cognitions. Behavioral problems are treated by social workers and behavioral trainers through coaching, observation, one-on-one conversations, and cognitive behavioral therapy. Another goal of the intervention is to enhance self-sufficiency in several life domains such as housing, finances, social network, mental health, substance abuse, and daytime activities. This is achieved through various educational courses (e.g., sport, cooking, culture), cognitive behavioral therapy, individually tailored coaching, as well as through offering regularity and structure. After the option to share a breakfast, all classes start at 9:30 and end at 14:30 or 15:30 for 4 days per week. Typical duration of the intervention is 5 to 6 months, which ends with participants successfully obtaining education or employment, or receiving a referral to specialized (mental) health care. A referral to specialized care only occurred if the participant displayed very severe mental health or very severe abuse-related problems and with consent of the participant. If the participant ends the intervention prematurely and without consent from the trainers, this is defined as treatment dropout.

### Predictors

Baseline variables were organized into four groups: (1) demographics and intelligence, (2) individual characteristics, (3), impulsivity, and (4) response inhibition and error processing.

The demographics and intelligence group consisted of ethnicity, education, and intelligence. The categorization of ethnicity was based on the Dutch definition of the Centraal Bureau voor de Statistiek (Statistics Netherlands) and included five categories: Western, Caribbean, Moroccan, Cape Verdean, and other non-Western. The largest ethnic minority groups in Rotterdam are people of Surinamese, Turkish, Moroccan, Antillean, and Cape Verdean origin, with the Surinamese community being the smallest and the Cape Verdean community the largest (Crul, Lelie, & Keskiner, 2019). However, due to the relatively small number of Turkish, Surinamese, and Antillean ethnic backgrounds in the current sample, final categorization included Western, Caribbean (e.g., Antillean and Surinamese ethnicity), Moroccan, Cape Verdean, and other non-Western (e.g., Turkish ethnicity). Education was categorized in primary only, junior secondary school, and senior secondary school. Intelligence was measured with four subscales of the Wechsler Adult Intelligence Scale–Third Edition (WAIS-III SF; digit symbol coding, information, block design, and arithmetic; Blyler, Gold, Iannone, & Buchanan, 2000) resulting in an estimated IQ score.

The individual characteristics included history of delinquency, regular use of cannabis and alcohol in years, aggression, psychopathy, and psychopathology. Previous research has shown that these individual characteristics are known to influence and predict treatment outcome. History of delinquency was assessed as the number of past offenses registered in the Research and Policy database Judicial Documentation (OBJD) by the Research Documentation Center (WODC) of the Ministry of Security and Justice in the Netherlands. Drug and alcohol use were assessed with the Measurements in the Addictions for Triage and Evaluation Questionnaire (MATE; Schippers, Broekman, Buchholz, Koeter, & van den Brink, 2010). Regular cannabis use was defined as the number of years of regular (i.e., weekly) cannabis use. Regular alcohol use was defined as the number of years of regular (i.e., weekly) alcohol use. Aggression was measured with the total score on the Reactive Proactive Aggression Questionnaire (RPQ; Cima, Raine, Meesters, & Popma, 2013; Raine et al., 2006). Psychopathy was assessed with the total score on the Youth Psychopathic Traits Inventory Short Version (YPI-sv; van Baardewijk, Andershed, Stegge, Nilsson, Scholte, & Vermeiren, 2010), and psychopathology was assessed with the Internalizing and Externalizing Problems score on the Adult Self-Report (ASR; Achenbach & Rescorla, 2003). The Internalizing Problems score included anxiety and depression, withdrawal, and somatic complaints. The Externalizing Problems included rule-breaking and aggressive behavior, as well as other social problems.

### Impulsivity

Impulse control was measured with the Dutch Barratt Impulsiveness Scale (BIS-11; Lijffijt, 2005; Patton, Stanford, & Barratt, 1995), a self-report questionnaire measuring impulsivity. The total score was used as a predictor.

### FMRI and behavioral measures of response inhibition

Response inhibition was measured with a Go/NoGo task previously used by Luijten et al. (2013). In short, participants are required to respond to vowels (i.e., Go trials) presented at 1 HZ, whilst refraining from responding when the presented letter is a repetition of the previous one (i.e., NoGo trials). In total, 817 Go and 110 NoGo trials were presented. ACC activity during the commission errors versus correct hits contrast was assessed in an a priori defined ROI (14 mm radius-sphere at *x* = 3, *y* = 24, *z* = 33; Aharoni et al., 2013). All images were acquired with a 3T GE Healthcare MRI scanner (The Discovery® MRI 750 3.0T, Milwaukee, MN, USA). BOLD T2-weighed axial images were acquired with echo planar imaging in 42 slices with a repetition time (TR) of 2,000 ms, echo time (TE) of 30 ms, flip angle (FA) of 80 degrees, field of view (FOV) 220 mm, and matrix size of 64 × 64 mm. A structural fast-spoiled gradient T1-weighted image was acquired in 180 sequential sagittal slices with a TR of 6.4 ms, TE of 2.8 ms, FA of 12 degrees, FOV of 240 mm, and the matrix size 240 × 240 mm. Imaging data were analyzed using Statistical Parametric Mapping 12 (SPM12; http://www.fil.ion.ucl.ac.uk/spm/). Preprocessing included the realignment and unwarping of all functional images. Next, the structural scan was coregistered to the mean T2*-weighted image and subsequently segmented. The images were normalized using the SPM T1-weighted MNI template and spatially smoothed with an 8-mm full-width half-maximum Gaussian filter. The four conditions, NoGo correct, NoGo incorrect, Go correct, and Go incorrect were modeled, and six movement parameters were added as covariates of no interest. ROI data for the ACC was extracted with the Marsbar toolbox for SPM (http://marsbar.sourceforge.net/). Lastly, three behavioral outcome measures were collected, percentage correct trials for NoGo trials (i.e., accuracy NoGo trials), average reaction time on correct Go trials, and average reaction time on incorrect NoGo trials.

### Electrophysiological and behavioral measures of error processing

Brain activity was recorded with a Biosemi ActiveTwo System amplifier to measure the EEG during an Eriksen flanker task previously used by Zijlmans et al. (2019). In short, participants responded to the middle letter in letter strings, HHHHH, SSSSS, HHSHH, SSHSS, by pressing the corresponding letter on the keyboard with their left or right index finger. Each string was presented for 52 ms, the maximum response time was 648 ms, and a stimulus was shown once every 1,450 ms. In total, 400 trials were presented per participant. Silver chloride (Ag/AgCl) electrodes were placed upon the scalp according to the International 10 –20 system, with two reference electrodes on the left and right mastoids. A sampling rate of 512 Hz and 24-bit analogue-to-digital conversion was used to digitized the signals. Offline filtering was done using a low cutoff of 0.15 Hz and a high cutoff of 30 Hz (24 dB/octave slope). To control for ocular artifacts, the vertical and horizontal electro-oculogram were assessed. Additional artifact rejection (±100 μV) was performed automatically. The −100–0-ms preresponse period served as baseline.

Error processing was measured with the response-locked ERN and Pe. For both indices, difference waves in mean activity across response conditions (incorrect minus correct) were calculated in a priori time intervals. The ERN was defined as the error-minus-correct difference wave in a 25–100 ms time window on the FCz electrode. The Pe was defined as the difference wave in a 250–400 time window on the Pz electrode. This was based on previous approaches typically used in ERN/Pe research (Brazil et al. 2009; Hajcak, Moser, Yeung, & Simons, 2005; Marhe et al., 2013; Olvet and Hajcak 2008; Zijlmans et al., 2019). Lastly, four behavioral outcome measures were collected, average reaction time for correct and incorrect trials, accuracy for total trials, and post error slowing effect (i.e., mean reaction time post error minus mean reaction time post correct response).

### Outcome measures

Outcome was assessed with two measures. The first outcome measure was treatment completion versus dropout as defined by DNK. DNK considered the treatment a success if the participant enrolled in education or employment, or if needed, if the participant was referred to specialized (mental) health care. According to DNK, a referral was an appropriate ending of the treatment, since they recognized and anticipated the specialized needs of the participant that could not be met at DNK itself. Treatment dropout was defined as dropout before finalizing the treatment and thus before enrollment in education or employment, or referral to specialized care, without mutual agreement between DNK and the participant. The second outcome measure was daytime activity versus no daytime activity a year after start of treatment at follow-up. Daytime activity was defined as part-time or full-time education, part-time or full-time employment, or other full-time daytime activities, such as full-time care for others, a sports membership, voluntary work, participation in a treatment program, internships, or starting one’s own business. It was possible for a participant to have more than one daytime activity. The different forms of daytime activities were used for the descriptive statistics, but not for analysis. A dichotomous variable for daytime activity (yes/no) was created and used for data analysis.

### Procedure

Data were collected by trained research assistants at the start of the treatment within the first 2 weeks (baseline), and 14 months later (follow-up). For the baseline measurement, questionnaires were administered in a maximum of two sessions in a period of 2 weeks, independent of the EEG and fMRI measurements. EEG measurements were assessed in one session at the Erasmus University Rotterdam, in the Erasmus Behavioral Lab of the Institute for Psychology. Participants were seated in a comfortable chair in a sound-attenuated room with dimmed lights. After explanation of the task by a trained researcher, participants started with a practice trial, followed by the experiment. The fMRI measurements were assessed in a different session than the EEG, in the Erasmus Medical Center Rotterdam. Participants received explanation from a trained researcher. The experiment started after a practice trial. The first outcome measure (treatment completion versus dropout) was registered by employees of DNK, at the end of treatment. The second outcome measure (daytime activities) was measured by research assistants at follow-up, 14 months after start of the treatment. For the follow-up measurement participants were contacted by phone, email, social media platforms (e.g., Facebook and WhatsApp), or by means of a house visit. The assessment was done either by phone, at DNK, or at a public place (in the latter case there were always two researchers present for safety purposes).

### Data analysis

To account for missing values (8.3% in total), multiple imputation for predictors with a maximum missingness of 30% was applied to impute to 30 complete sets (White, Royston, & Wood, 2011). Little’s Missing Completely At Random (MCAR) test was employed; the data were MCAR (*x* ^2^ = 100.223, *df* = 107, *p* = .66). Logistic regressions were employed to assess the predictive value of background, behavioral, and neurobiological factors on the two treatment outcome measures, completion versus dropout and daytime activity versus no daytime activity at follow-up. The predictor variables were tested for normality of distribution, linearity, multicollinearity, and independence of errors. Box–Tidwell transformations were used to test the assumption of linearity between the continuous predictors and the logit of the dependent variables. Linearity was not violated. In addition, multicollinearity was tested by inspecting variance inflation factors (VIF). VIF values greater than 10 indicate a multicollinearity problem (Myers, 1990). Most VIF values did not exceed 10, except for average reaction time on correct flanker trials and average reaction time on incorrect flanker trials. As both variables measure reaction time on the flanker task, it is not uncommon to discover dependency between these variables. However, these results should still be interpreted with caution. Lastly, independence of errors was tested by looking at the residuals. This assumption was not violated. A Western ethnic background and primary education only were used as reference indicators for the categorical variables. Two hierarchical regression analyses were applied to examine the predictive power of the behavioral and neurobiological variables over and above the demographic variables and the individual characteristics. The predictors were forced into the model in the following sequence: (1) demographics and intelligence, (2) individual characteristics, (3) impulsivity, and (4) response inhibition and error processing. The level of significance was set at *p* = .05. The Results section will first report the descriptive statistics, then the outcome of the logistic regression on treatment completion versus dropout, and lastly the outcome of the logistic regression on daytime activities versus no daytime activities. Individual contribution of the predictors per logistic regression were also examined.

## Results

**Fig. 1:**
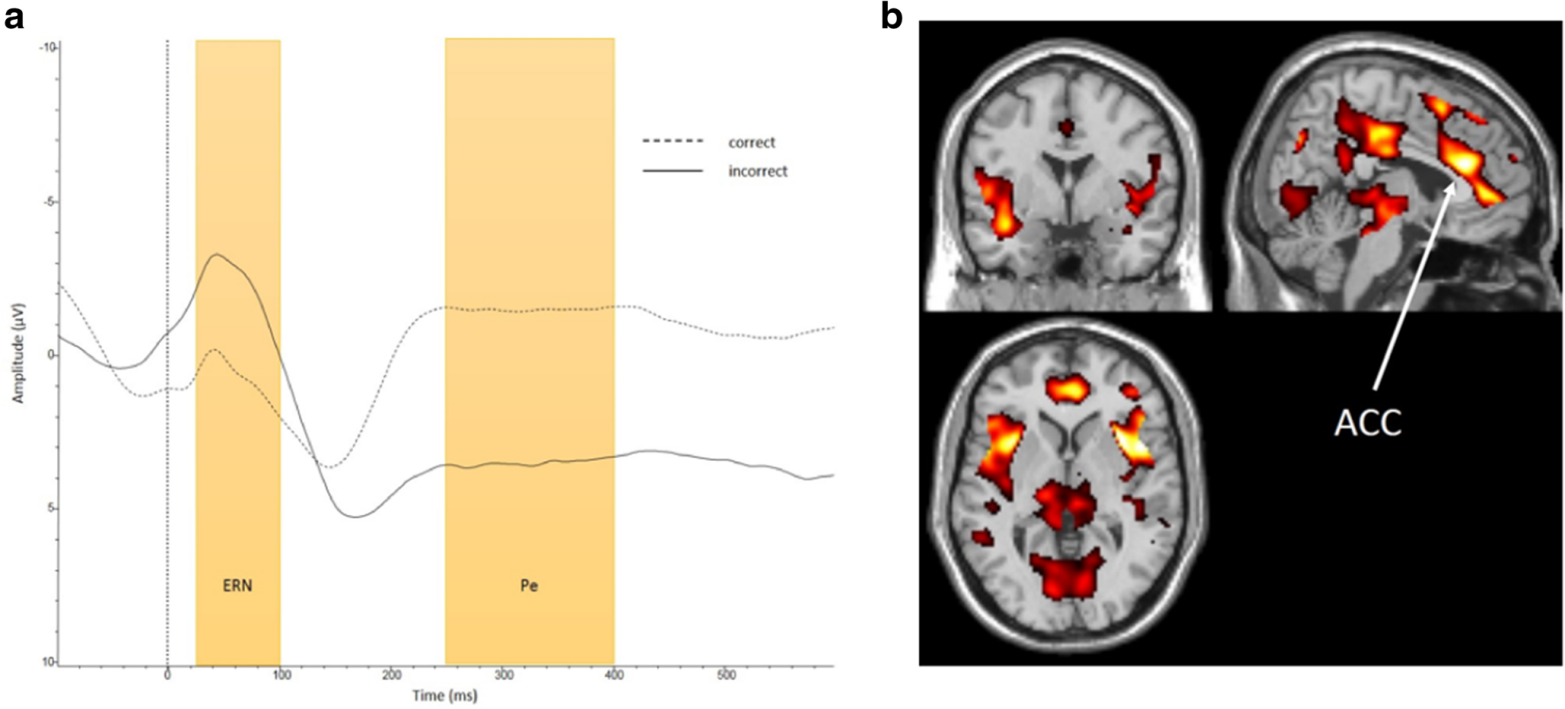
**a** Electroencephalographic waveforms in response to correct and incorrect trials. ERN = error-related negativity; Pe = error positivity. **b** Whole-brain family-wise error corrected hemodynamic activity during the commission errors versus correct hits contrast, *x* = 49, y = 64, *z* = 38. ACC = anterior cingulate cortex. (Color figure online)

Descriptive statistics (*M, SD*,*%*) of all predictor variables are shown in Table 1. Group-level average ERPs and fMRI activation patterns are displayed in Fig. 1.

**Table 1:** Descriptives of all predictors

Variable	*Mean / N*	*SD / %*
Ethnicity		
Western	18	17.1
Caribbean	41	39.0
Moroccan	20	19.0
Cape Verdean	7	6.7
Other non-Western	19	18.1
Education		
Primary only	46	43.8
Junior secondary	38	36.2
Senior secondary	21	20.0
IQ	82.3	9.8
Number of past offenses < treatment start	4.5	4.4
Regular use of cannabis in years	4.3	3.7
Regular use of alcohol in years	2.3	3.6
RPQ total	16.3	7.6
ASR Internal	72.5	24.1
ASR External	69.2	23.8
YPI-sv total	33.1	7.8
BIS-11 total	64.1	8.9
ACC activity during response inhibition	2.8	2.4
Reaction time GO trials	409.9	53.6
Reaction time NOGO trials	392.3	81.5
Accuracy NOGO trials	.5	0.1
ERN flanker task	-4.8	4.6
Pe flanker task	5.8	5.2
Reaction time correct flanker trials	450.3	74.0
Reaction time incorrect flanker trials	406.0	76.0
Accuracy flanker trials	.8	0.1
Post error slowing effect	43.0	38.4

*Note.* IQ = intelligence quotient; RPQ = Reactive Proactive Questionnaire; ASR = Adult Self-Report; YPI-sv = Youth Psychopathic Traits Inventory–Short Version; BIS-11 = Barratt’s Impulsivity Scale; ACC = anterior cingulate cortex; ERN = error-related negativity; Pe = error positivity. *N* = 105 for all variables

### Outcome measures

Directly posttreatment, 61 (58.0%) participants were successfully enrolled into either education (17.1%) or work (29.5%), or referred to other care (11.4%). Information on treatment success or failure was missing for one participant. The remaining 43 (41.0%) participants were dropouts.

A year after start of treatment, 93 participants responded to the follow-up assessment. Twelve participants were lost to attrition. A total of 49 (46.7%) participants had found one or more daytime activities, of which 12 participants were previously classified as treatment dropouts. An overview of the different daytime activities can be found in Table 2. On average, participants remained in treatment for 171.88 days until treatment success (*N* = 61) or 121.08 days until treatment dropout (*N* = 43).

**Table 2:** Overview of daytime activities a year after start of treatment

Daytime activities	*N*	*%*
Work	26	24.8
Education	18	17.1
Sports membership	7	6.7
Care for others	4	3.8
Treatment program	5	4.8
Voluntary work	6	5.7
Other	5	4.8
No daytime activity	44	41.9
Nonresponse	12	11.4

*Note. N* = 105

Results of the first hierarchical regression predicting treatment completion versus dropout are shown in Table 3. The model containing only demographics and intelligence (*x* ^*2*^ = 0.709, *df* = 7, *p* = .664) did not significantly predict treatment outcome. Adding the individual characteristics did not significantly improve the model (*x* ^*2*^ = 0.628, *df* = 14, *p* = .844), nor did adding impulsivity (*x* ^*2*^ = 0.639, *df* = 15, *p* = .844). The overall model containing all predictors including error processing and response inhibition did not significantly predict treatment outcome (*x* ^*2*^ = 0.502, *df* = 25, *p* = .981). Controlling for all other predictors, none of the individual predictors reached significance.

**Table 3:** Results of hierarchical regression predicting treatment outcome

	*B*	*SE (B)*	*p*	95% CI *B*	
				*Lower*	*Upper*
*Block 1*					
Constant	.693	2.009	.730	.039	102.688
Western	Ref.				
Caribbean	.314	.608	.605	.416	4.505
Moroccan	−.469	.667	.482	.169	2.313
Cape Verdean	1.357	1.220	.264	.356	42.549
Other non-Western	.313	.709	.659	.341	5.495
Primary only	Ref.				
Junior secondary	−.555	.568	.328	.189	1.745
Senior secondary	.047	.471	.921	.417	2.635
IQ	−.005	.022	.839	.953	1.040
*Block 2*					
Constant	.749	2.410	.756	.019	237.910
Western	Ref.				
Caribbean	.208	.657	.751	.340	4.465
Moroccan	−.880	.740	.234	.097	1.770
Cape Verdean	1.475	1.316	.265	.329	57.692
Other non-Western	.261	.740	.724	.304	5.540
Primary only	Ref.				
Junior secondary	−.489	.598	.414	.190	1.982
Senior secondary	.035	.499	.945	.389	2.756
IQ	.007	.024	.771	.961	1.055
History of delinquency	.023	.053	.659	.923	1.135
Regular use of cannabis in years	.036	.068	.598	.907	1.185
Regular use of alcohol in years	−.128	.075	.087	.760	1.019
RPQ total	−.003	.034	.926	.933	1.066
ASR internal	.004	.012	.771	.979	1.028
ASR external	−.010	.014	.478	.963	1.018
YPI-sv total	−.011	.035	.762	.923	1.060
*Block 3*					
Constant	1.822	.2697	.499	.031	1222.183
Western	Ref.				
Caribbean	.197	.661	.766	.333	4.443
Moroccan	−.982	.755	.194	.085	1.647
Cape Verdean	1.448	1.314	.255	.325	56.250
Other non-Western	.198	.744	.790	.284	5.235
Primary only	Ref.				
Junior secondary	−.602	.619	.331	.163	1.842
Senior secondary	.018	.502	.972	.381	2.722
IQ	.009	.024	.720	.962	1.057
History of delinquency	.031	.054	.563	.928	1.146
Regular use of cannabis in years	.036	.068	.594	.907	1.186
Regular use of alcohol in years	−.138	.076	.071	.750	1.012
RPQ total	−.003	.034	.928	.932	1.066
ASR internal	.004	.012	.722	.980	1.029
ASR external	−.005	.015	.731	.966	1.024
YPI-sv total	−.001	.038	.982	.928	1.076
BIS-11 total	−.029	.034	.379	.909	1.037
*Block 4*					
Constant	1.556	3.578	.664	.004	5,269.558
Western	Ref.				
Caribbean	−.093	.798	.907	.191	4.355
Moroccan	−1.359	.840	.106	.050	1.332
Cape Verdean	1.531	1.402	.277	.299	.72.840
Other non-Western	.081	.863	.925	.200	5.886
Primary only	Ref.				
Junior secondary	−.641	.687	.351	.137	2.025
Senior secondary	.102	.563	.856	.367	3.339
IQ	.003	.030	.926	.946	1.063
History of delinquency	.057	.064	.368	.935	1.200
Regular use of cannabis in years	.044	.081	.592	.891	1.225
Regular use of alcohol in years	−.174	.090	.052	.705	1.002
RPQ total	−.007	.038	.863	.922	1.071
ASR internal	.003	.014	.829	.975	1.032
ASR external	−.005	.016	.781	.964	1.028
YPI-sv total	−.004	.044	.930	.913	1.087
BIS-11 total	−.028	.038	.466	.904	1.047
ACC activity during response inhibition	.156	.129	.229	.907	1.506
Average RT GO	.000	.001	.558	.998	1.001
Average RT NOGO	.000	.001	.886	.999	1.001
Accuracy NOGO	1.576	2.100	.453	.078	298.998
ERN flanker	.006	.068	.929	.881	1.149
Pe flanker	−.016	.063	.801	.870	1.113
Average RT correct flanker	.012	.013	.352	.986	1.039
Average RT incorrect flanker	−.009	.012	.451	.969	1.014
Total accuracy flanker	−.722	3.587	.841	.000	550.019
Post error slowing	.005	.009	.634	.986	1.023

*Note*. Nagelkerke’s *R*^2^ for Block 1 = .064, Nagelkerke’s *R*^2^ for Block 2 = .062, Nagelkerke’s *R*^2^ for block 3 = .133, Nagelkerke’s R2 for Block 4 = .209. IQ = intelligence quotient; RPQ = Reactive Proactive Questionnaire; ASR = Adult Self-Report; YPI-sv = Youth Psychopathic Traits Inventory–Short Version; BIS-11 = Barratt’s Impulsivity Scale; ACC = anterior cingulate cortex; ERN = error-related negativity; Pe = error positivity

Results of the second hierarchical regression predicting daytime activity versus no daytime activity at follow-up are shown in Table 4. The model containing only demographics and intelligence (*x*^*2*^ = 1.654, *df* = 7, *p* = .115) failed to reach statistical significance, as did the models including individual characteristics (*x* ^*2*^ = 1.695, *df* = 14, *p* = .052) and impulsivity (*x* ^*2*^ = 1.701, *df* = 15, *p* = .064). The overall model including error processing and response inhibition also did not significantly predict whether the participant engaged in a daytime activity at follow-up (*x*^*2*^ = 1.519, *df* = 25, *p* = .059). However, when controlling for all other predictors, the ACC activity during the Go/NoGo contrast (*p* = .026, odds ratio = 0.408), regular alcohol use in years (*p* = .041, odds ratio = -0.258), the Internalizing Problem Score on the ASR (*p* = .014, odds ratio = 0.054), and a Moroccan ethnic background versus Western ethnic background (*p* = .004, odds ratio = -4.005) all significantly predicted whether a participant engaged in a daytime activity. In other words, increased ACC activity during response inhibition, decreased years of regular alcohol use, and an increased score on internalizing problems all resulted in higher odds on daytime activity. In addition, participants with a Moroccan ethnic background had lower odds on daytime activity at follow-up compared with participants with a Western ethnic background. Note that a Moroccan ethnic background versus Western ethnic background and the Internalizing Problem Score were also significant in the previous blocks (1, 2, 3). In contrast, regular alcohol use in years only reached significance in the last block.

**Table 4:** Results of hierarchical regression predicting daytime activity

	*B*	*SE (B)*	*p*	95% CI *B*	
				*Lower*	*Upper*
*Block 1*					
Constant	−.493	2.163	.820	.009	42.342
Western	Ref.				
Caribbean	−.326	.658	.620	.199	2.619
Moroccan	−1.965	.803	.014	.029	.677
Cape Verdean	−1.553	1.050	.139	.027	1.657
Other non-Western	−.269	.744	.718	.178	3.287
Primary only	Ref.				
Junior secondary	.658	.632	.298	.559	6.671
Senior secondary	.588	.508	.247	.665	4.876
IQ	.010	.024	.666	.964	1.060
*Block 2*					
Constant	−1.943	2.914	.505	.000	43.304
Western	Ref.				
Caribbean	−.329	.761	.665	.162	3.197
Moroccan	−2.582	.953	.007	.012	.490
Cape Verdean	−1.550	1.145	.176	.022	2.004
Other non-Western	−.226	.849	.790	.151	4.212
Primary only	Ref.				
Junior secondary	.719	.684	.293	.537	7.852
Senior secondary	.716	.572	.210	.667	6.276
IQ	.027	.028	.334	.973	1.085
History of delinquency	−.034	.072	.641	.839	1.114
Regular use of cannabis in years	.131	.085	.121	.966	1.346
Regular use of alcohol in years	.−1.42	.085	.095	.734	1.025
RPQ total	−.030	.040	.458	.898	1.050
ASR internal	.035	.015	.018	1.006	1.065
ASR external	−.026	.017	.117	.943	1.007
YPI-sv total	−.005	.041	.895	.918	1.078
*Block 3*					
Constant	−.312	3.174	.922	.001	368.065
Western	Ref.				
Caribbean	−.393	.772	.610	.148	3.066
Moroccan	−2.777	.980	.005	.009	.425
Cape Verdean	−1.618	1.151	.160	.021	1.893
Other non-Western	−.407	.865	.638	.122	3.627
Primary only	Ref.				
Junior secondary	.604	.702	.390	.462	7.245
Senior secondary	.678	.579	.242	.633	6.130
IQ	.032	.029	.262	.976	1.092
History of delinquency	−.025	.074	.737	.844	1.127
Regular use of cannabis in years	.130	.085	.126	.964	1.346
Regular use of alcohol in years	−.152	.086	.077	.725	1.017
RPQ total	−.029	.040	.467	.897	1.051
ASR internal	.038	.015	.012	1.008	1.069
ASR external	−.019	.018	.284	.948	1.016
YPI-sv total	.011	.045	.799	.926	1.105
BIS-11 total	−.050	.040	.207	.879	1.028
*Block 4*					
Constant	1.844	4.797	.701	.001	77018.971
Western	Ref.				
Caribbean	−1096	1.070	.306	.041	2.728
Moroccan	−4.005	1.399	.004	.001	.283
Cape Verdean	−.956	1.391	.492	.025	5.866
Other non-Western	−.678	1.236	.583	.045	5.722
Primary only	Ref.				
Junior secondary	.646	.912	.479	.319	11.403
Senior secondary	1.223	.769	.112	.752	15.351
IQ	.033	.039	.406	.956	1.116
History of delinquency	.063	.102	.540	.871	1.301
Regular use of cannabis in years	.192	.118	.105	.961	1.527
Regular use of alcohol in years	−.258	.126	.041	.603	.989
RPQ total	−.061	.053	.249	.849	1.044
ASR internal	.054	.022	.014	1.011	1.102
ASR external	−.022	.022	.304	.937	1.020
YPI-sv total	−.004	.065	.951	.876	1.132
BIS-11 total	−.039	.056	.494	.861	1.075
ACC activity during response inhibition	.408	.183	.026	1.050	2.155
Average RT GO	.000	.001	.687	.998	1.003
Average RT NOGO	−.001	.001	.231	.997	1.001
Accuracy NOGO	1.451	2.737	.596	.020	918.518
ERN flanker	.100	.099	.308	.911	1.342
Pe flanker	.083	.093	.368	.906	1.304
Average RT correct flanker	.036	.020	.072	.997	1.078
Average RT incorrect flanker	−.033	.018	.062	.934	1.002
Total accuracy flanker	−5.988	5.062	.237	.000	51.249
Post error slowing	.006	.013	.628	.981	1.033

*Note*. Nagelkerke’s *R*^2^ for Block 1 = .156, Nagelkerke’s *R*^2^ for Block 2 = .312, Nagelkerke’s *R*^2^ for Block 3 = .332, Nagelkerke’s R2 for Block 4 = .518. IQ = intelligence quotient; RPQ = Reactive Proactive Questionnaire; ASR = Adult Self-Report; YPI-sv = Youth Psychopathic Traits Inventory–Short Version; BIS-11 = Barratt’s Impulsivity Scale; ACC = anterior cingulate cortex; ERN = error-related negativity; Pe = error positivity

## Discussion

The present study addressed the predictive value of neurobiological and neurobehavioral measures of cognitive control in relation to multiproblem young adults’ treatment completion and engagement in daytime activities at follow-up. The results of the overall models showed that individual characteristics (psychological, social, criminal) were not associated with treatment completion or daytime activities at follow-up. Most pertinent to this study, is that the addition of error processing and response inhibition did not change the results, meaning that both the original model and the complete model containing the neurobiological and neurobehavioral measures of cognitive control did not reach significance. However, when controlling for all other predictors, activity in the ACC during response inhibition, regular use of alcohol in years, internalizing problems, and ethnicity all significantly predicted whether a participant engaged in a daytime activity.

In the current paper, cognitive control was not associated with treatment completion versus dropout. This is in line with a study amongst aggressive forensic psychiatric outpatients, where behavioral response inhibition could not distinguish between treatment completers and dropouts (Smeijers, Bulten, Buitelaar, & Verkes, 2018). Likewise, in a study on substance abusers (Moriyama et al., 2002), treatment effectiveness (e.g., resumed substance use after treatment or not) could not be predicted with neuropsychological tests of cognitive control. As opposed to these studies and the current results, aberrant error processing as indicated by a smaller ERN (Marhe et al., 2013) and a larger Pe (Steele et al., 2017) has been previously predictive of treatment completion in adult substance abusers. One explanation for the difference in results on error processing might be the difference in patient population. The current heterogeneous population suffered from a plethora of problems of varying severity, including, but not limited to, history of delinquency, behavioral and psychological problems, no daytime activities, frequent substance use, and no or low income (Luijks et al., 2017; van Duin et al., 2017; van Duin et al., 2018; Zijlmans et al., 2019; Zijlmans et al., 2018). It is possible that aberrant error processing reflects a deficit in substance users and thus efficiently discriminates between substance abusers and nonabusers in treatment outcome, but this effect does not apply to heterogeneous populations such as the current sample. Another possible explanation may be the difference in age, as the current study focused on young adults rather than adults. Some research suggests the Pe increases with age (Grammer, Carrasco, Gehring, & Morrison, 2014), although other studies do not support these findings (Davies, Segalowitz, & Gavin, 2004; Santesso, Segalowitz, & Schmidt, 2006). Likely, the age range of the participants in the current study was too small to establish any age-related differences, thus studies including more age-related heterogeneity are warranted to uncover such effects.

Notwithstanding the result of the overall model failing to predict daytime activity at follow-up, we found a direct association with ACC activity during a response inhibition task. Increased ACC activity during response inhibition was associated with higher odds of involvement in daytime activities 1 year after start of treatment. This suggests that adequate inhibitory control (Kerns et al., 2004) at baseline is associated with a positive outcome (e.g., participation in daytime activities) a year later at follow-up. This is in line with a previous study, where increased ACC activity has been related to lower instance of rearrests in adult offenders (Aharoni et al., 2013). It might therefore be beneficial to increase cognitive control by means of interventions that modulate ACC activity. This in turn could lead to better outcomes. Previous studies suggest a positive effect of physical activity on cognitive control, through increased neural efficiency (Erickson, Hillman, & Kramer, 2015) in the prefrontal cortex and ACC (Voss et al., 2011). Furthermore, a reduction in ACC activity is related to better treatment engagement (Devito et al., 2017). It is thus possible that interventions targeting ACC activity, for example through physical activity, could potentially increase neural efficiency (thus reducing activity) in cognitive control-related regions, and subsequently improve cognitive control. This could in turn result in more positive outcomes at follow-up, but more longitudinal studies are needed to explore this.

The question arises as to why the current measurements of cognitive control failed to predict treatment completion versus dropout, yet ACC activity during response inhibition (a marker of cognitive control), did predict participation in daytime activities at follow-up. It is possible that higher level cognitive abilities such as response inhibition and error processing are more important for retaining, rather than acquiring, successful reintegration into society. This could be due to the diminishing degree of guidance as time elapses. As treatment completion is measured in terms of reentry into society through the means of education or work, the intervention facilitates and structures this transition as much as possible. However, after treatment completion or dropout, the facilitation and structure from the intervention is discontinued, and participants must rely more on their own individual abilities, such as cognitive control. This could explain why some measurements of cognitive control, such as ACC activity during response inhibition, are more sensitive to predicting daytime activities at follow-up than to predicting treatment completion directly posttreatment. Better aftercare or more tailored aftercare—for example, more structured aftercare for participants with less cognitive control—could possibly aid participants in retaining successful reintegration into society through continued participation in daytime activities.

Besides brain activity during response inhibition, more years of regular (i.e., weekly) alcohol use was also related to lower odds on daytime activity participation at follow-up. Regular alcohol use impairs inhibition and judgment (Lee & Snape, 2008), which could result in socially undesirable behavior or other forms of social dysfunction, diminishing the likelihood to acquire or retain daytime activities. This is supported by a prospective cohort study in adults relating high alcohol consumption and problem drinking to adverse labor market transitions, such as a lower chance on finding a new job after being unemployed as well as a higher chance of becoming unemployed (Jørgensen et al., 2019). Similarly, another study in young university students found an association between high levels of alcohol consumption and low academic performance as well as low mental health outcomes (Tembo, Burns, & Kalembo, 2017). In short, these findings suggest that regular alcohol use has a negative impact on daytime activities such as work and school.

Additionally, internalizing, but not externalizing problems at baseline were associated with daytime activities at follow-up. This is partly in line with previous literature, where both increased internalizing and externalizing problems were related to negative outcomes such as poorer work performance and poorer academic achievement (Korhonen, Luoma, Salmelin, Siirtola, & Puura, 2018; Mordre, Groholt, Sandstad, & Myhre, 2012; Narusyte, Ropponen, Alexanderson, & Svedberg, 2017). In contrast, the current study found an association between greater internalizing problems and a positive outcome (i.e., better odds on daytime activity). Internalizing problems such as depression and anxiety are covert, often overlooked behaviors (Miller & Jome, 2010), especially in young adults with comorbid externalizing problems (Hankin et al., 2016). Internalizing problems are therefore sometimes described as a secret illness (Reynolds, 1992). In contrast, externalizing problems are mostly overt and more easily detected behaviors due to their disruptive nature (Forns, Abad, & Kirchner, 2011). It may be that participants with more severe or pronounced internalizing problems showed less externalizing behavior. This is supported by studies suggesting internalizing problems could act as a constraining factor in the development of externalizing behavior, possibly related to withdrawal or inhibition (Masten et al., 2005; Mesman, Bongers, & Koot, 2001; Moffitt, Caspi, Harrington, & Milne, 2002). Speculatively, it is also possible that individuals with more severe or obvious internalizing problems were treated more leniently regarding their disruptive behavior, causing them to acquire and retain more daytime activities than those with less internalizing problems.

Lastly, participants with a Moroccan ethnicity had lower odds on daytime activities compared with participants with a Western ethnicity. In line with this result, persons of Moroccan origin are often represented as the ethnic group with the least adequate reintegration into the Dutch society (Dagevos, Gijsberts, & van Praag, 2003; Roggeband & van der Haar, 2018) compared with other ethnic minorities. Moreover, participants of Moroccan ethnicity face more problems compared with other ethnic minorities, such as underreporting of mental health problems (Stronks, Ravelli, & Reijneveld, 2001; Uiters, Devillé, Foets, & Groenewegen, 2006), higher perceived discrimination (Dagevos et al., 2003; Roggeband & van der Haar, 2018), and more negative stereotyping (van Craen, Vancluysen, & Ackaert, 2007). Thus, it is plausible that Moroccan migrants are more sensitive to certain treatment characteristics, such as sharing of worldview, empathy, expertise, and ethnic matching in caregivers and participants (Knipscheer & Kleber, 2004). The current study did not include such treatment characteristics in the analysis, thus future studies should examine their possible effect on the association between daytime activities and cognitive control in different ethnic groups.

A relatively large number of predictors were used in the current study, which could explain why most of the individual measures did not reach significance, except for ACC activity, regular alcohol use in years, internalizing problems, and ethnicity. Nonetheless, the current predictors were included because the model adopted a biopsychosocial model in which the joint contribution of various predictors was examined. It is also possible that treatment characteristics, such as the combination of techniques from cognitive-behavioral therapy (CBT) and practical support play a role in this relationship. It is assumed that successful use of CBT relies on the adequate use of cognitive control (Andrew James, Reichelt, Carlsonn, & McAnaney, 2008; Goodkind et al., 2016; Mohlman & Gorman, 2005), but the association with other forms of intervention, such as practical support or lessons, remains unclear. Therefore, it is possible that the current multimodal treatment relies on a combination of cognitive control and individual abilities such as treatment motivation (Walton, 2015). This could imply that cognitive control is only associated with treatment outcome when controlling for additional individual abilities, or that cognitive control is not associated with the current multimodal treatment. The current study included other relevant individual characteristics such as psychological state and IQ, but did not include individual treatment characteristics such as treatment motivation. Future studies should include other individual characteristics to uncover any effect of the individual on the association with treatment outcome.

The current study is not without limitations. Firstly, self-report questionnaires were used to measure the individual characteristics of the participants, and thus their answers could be biased or completed in a socially desirable manner. Secondly, the low average IQ of the current sample (mean = 82) and low average educational level (20% finished secondary school) could influence the results due to lack of understanding of the tests, although post hoc analysis revealed no relationship between IQ and any of the predictors or outcome measures. Thirdly, due to small sample sizes per activity, the current study did not distinguish between the different types of daytime activities. Future research should replicate this study with larger sample sizes, to determine any effect of the type of activity on cognitive control. Previous research has established the critical role of cognitive control in education and work (e.g., Diamond, 2013), yet for other daytime activities such as sports membership or caring for others this remains unclear. It is thus possible that different types of daytime activities require different amounts of cognitive control, resulting in different outcomes. Lastly, DNK considered the treatment as completed after enrollment in education or employment, or after referral to appropriate (mental) health care. A referral to health care could be seen as a different outcome compared with education and employment, which could possibly threaten the generalizability of the current study. However, the main goal of the treatment was participation in education or employment. A referral to specialized care only occurred if the participant displayed too severe mental health or too severe abuse-related problems and with consent of the participant. If a participant received a referral, the treatment at the current intervention was (temporarily) discontinued, thus the treatment was seen as completed. Future research could also focus on both children, adolescents, and adults to distinguish any age-related effect on the association with cognitive control and treatment outcome, as it is possible that different age groups display a different relation between cognitive control and treatment outcome. Another recommendation could be to monitor and distinguish the amount of aftercare participants receive (e.g., from the intervention, work, or education), to examine if this could explain different associations of cognitive control directly post-treatment versus at follow-up. Lastly, other approaches might be able to examine whether cognitive control has a population-related association with a substance abuse population by comparing the effect of cognitive control in a substance abuse population versus other populations showing externalizing problems such as antisocial behavior.

Biomarkers (including neurobiological markers) provide objective and measurable indices that could aid in the individualization of treatment and the prediction of antisocial behavior. However, several ethical concerns have been raised about the use of such markers for the prediction of antisocial behavior (Jurjako, Malatesti, & Brazil, 2019). These include the extrapolation of group-level information to gain knowledge on an individual level (Dawid, 2017), large error margins in risk estimates (Monahan, 2014), differences in the conceptualization of behavior between the legal system and science (Buckholtz & Faigman, 2014; Francken & Slors, 2018), and the heterogeneous, symptomatic conceptualization of most psychiatric disorders (Jurjako et al., 2019). First, the legal system is mostly interested in individual propensities, whereas scientific research commonly uses group average data. The use of information on group level could obscure detection of individual differences, which could lead to biases. Second, the relatively large error margins in risk-assessment tools lower the certainty for an individual’s propensity for future violent behavior. Third, legal constructs are often prone to different conceptualizations than those used in biological processes. This could lead to wrong impressions about reliability and relevance of the biomarker information, as not all legal constructs are directly transposable to the biological processes. Lastly, most psychiatric disorders are conceptualized as heterogeneous dimensional constructs. This current taxonomy relies mostly on behavior and, just as with the legal constructs, not all behavior is transposable to biological processes, which could affect the predictive value of biomarkers on their own. This is in line with the biopsychosocial model in which combinations of biological, psychological, and social factors should be taken into account in the prediction of antisocial behavior (Popma & Raine, 2006). Jurjako et al. (2019) conclude that these issues are important to consider, but they do not argue against the use of biomarkers in the prediction of antisocial behavior; rather, they advise to take caution when using biomarkers.

In conclusion, the overall models containing cognitive control as measured by neurobiological and neurobehavioral factors did not predict treatment completion or daytime activities at follow-up in multiproblem young adults. However, ACC activity during a response inhibition task did predict daytime activities when controlling for other measurements of cognitive control and individual characteristics, suggesting a possible role for inhibitory control.
